# Assigning Viscosity Values in the Glass Softening Temperature Range

**DOI:** 10.3390/ma16041596

**Published:** 2023-02-14

**Authors:** Miguel O. Prado, Franco E. Benedetto

**Affiliations:** 1 Comisión Nacional de Energía Atómica, San Carlos de Bariloche 8400, Río Negro, Argentina; 2 Consejo Nacional de Investigaciones Científicas y Técnicas, San Carlos de Bariloche 8400, Río Negro, Argentina

**Keywords:** viscosity, glass, glass transition temperature, hot stage microscopy

## Abstract

A new optical method for assigning glass viscosity values in the softening temperature range is presented. In this method, an irregular particle, a few millimeters in size, laying on an alumina plate, is heated up to temperature *T*, and then remains at this temperature. *T* should be within the softening temperature range of the glass. There are no external applied shear stresses, the only acting shear forces are those coming from the particle’s own surface energy. At the fixed temperature *T*, the surface free energy of the sample decreases by viscous flow while its shape evolves from a polyhedron or irregular shape towards a spherical or rounded shape. This shape evolution is recorded using a photographic camera. From each image, the sample’s roundness is determined, obtaining a characteristic time *τ* from the roundness against time. Simultaneously, using the available software, a value for the viscosity *η* was calculated, at temperature *T*, allowing for building sets of *T*, *τ*, *η*, namely three data values. Accordingly, if *T*, *τ* are considered as independent variables, a master function *η* = *η* (*T*, *τ*) can be built. Now, if we measure *T*, *τ* data on a glass of an unknown viscosity, the master function makes it possible to assign a *η* value. When incipient crystallization or liquid–liquid phase separations are present, effective viscosity values are obtained. This method requires a high temperature microscope, as well as tridimmensional samples with a few cubic millimeters of volume. Each isothermal *τ* determination can take from minutes to several hours. We tested the method with two glasses of known viscosity values: borosilicate glass (VG98) and alumimoborosilicate glass (SG7), both of which are used for radioactive waste immobilization and have assigned log(*η*) values between 6 and 7.3 with *η* in Pa s. The discrepancy between the log(*η*) values assigned here and those values fitted with a VFT function on the values measured for the SG7 and VG98 glasses were within ±14%.

## 1. Introduction

**Figure 1:**
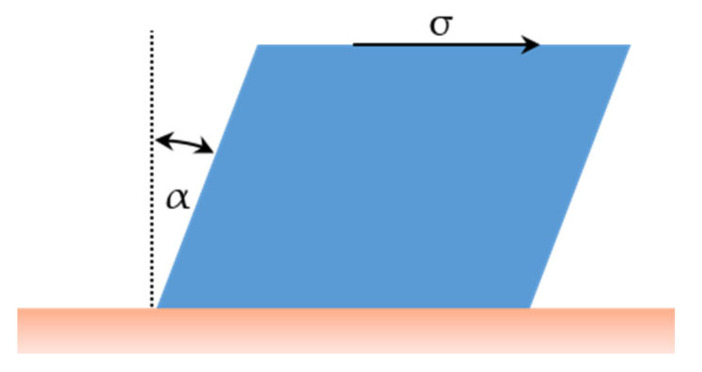
Quantities involved in viscosity definition.

When heating glass above its glass transformation temperature range, it goes from solid glass to unstable supercooled liquid. Among the fundamental quantities that characterize the supercooled liquid and liquid behavior, viscosity is of particular interest to technologists and geologists. Viscosity can be defined as the proportionality constant between the shear stress *σ* applied to the opposite faces of a glass cube and its angular deformation rate $\frac{d\alpha }{dt}$, as stated in Equation (1) and represented in Figure 1 [1,2], at a fixed pressure and temperature.

(1) $$\sigma =\eta \;\frac{d\alpha }{dt}$$

As can be seen from Equation (1), *η* units are measured in Pa s. At around the glass transition temperature *T_g_*, the viscosity value for glasses is around 10^12.5^ Pa s [3,4]. After heating, it decreases to 10^0^ Pa s until reaching melting temperature. The approximate viscosity values for glass forming operations can be found in [5]. Such a strong variation in its value requires that different techniques must be applied for its measurement. Among others, in viscosity values ranging 10^5^–10^12^ Pa s, parallel plate, penetration, fiber elongation, or beam bending methods are used. Stokes’ falling sphere/bubble rise methods are usually used at a low viscosity of <10^4^ Pa s. At viscosity values <10^6^ Pa s, the rotating concentric cylinders technique is the standard [6,7]. Several models can be used to obtain theoretical viscosity values as a function of composition and temperature. However, these models have composition and temperature restrictions, and in the case of being proper models for the glass system of interest, considerable differences among the predicted values from different models can be found [8,9,10,11,12,13].

Regarding the dependence of viscosity on temperature, certain glass obeys an Arrhenius law, $\eta =\eta_{0}e^{\frac{A}{T}}$, over a wide temperature range, which are known as strong glasses or as silica glass [14]. Fragile glasses obey the Vogel–Fulcher–Tamman (VFT) empirical equation with three temperature-independent parameters, namely *η*_0_, A, and *T*_o_, which describe the viscosity dependence on temperature (Equation (2)) within 10 orders of magnitude, while the glass structure is changing. VFT is used in the viscosity range from 10^3^ to 10^12^ Pa s, which includes intermediate viscosity values, namely at the softening range, where the present paper applies. However, it fails for *η* < 10^2^ Pa s, when the melt is almost fully depolymerized and behaves as an ordinary liquid [15].

(2) $$\eta =\eta_{0}e^{\frac{A}{T-T_{0}}}$$

Another fundamental property of glasses is relaxation. There is structural relaxation, stresses relaxation, and residual relaxation. According to Lancelotti et al. [16], following Scherer [17] and Doss et al. [18], structural relaxation is “the response of a material subjected to an isothermal hold measured through observable changes in the material’s properties […] due to structural rearrangements over time”. Stress relaxation is “the time-dependent response of the stress developed within a material subjected to a mechanical strain”. Residual relaxation is that due to the stresses generated by different cooling rates on the same sample.

When structural relaxation is present at a fixed pressure and temperature, a simple exponential decay behavior does not correctly describe most of the processes. One of the simplest equations for relaxation kinetics is the Kohlrausch equation [19,20,21]. See Equation (3).

(3) $$\varphi \left(t,T,T_{f}\right)=e^{{\left(\frac{-t}{\tau_{K}\left(T,T_{f}\right)}\right)}^{\beta \left(T,T_{f}\right)}}$$

where *φ* is the relaxation parameter, which takes values $\varphi \left(0,T,T_{f}\right)=1$ and $\varphi \left(\infty ,T,T_{f}\right)=0$. $T_{f}$ is the fictive temperature at *t* = 0, $\tau_{K}$ is the decay structural relaxation time, and *β* is the Kohlrausch exponent.

However, for stress relaxation, recently, Jarauta et al. [22] used the Prosperetti [23] theoretical results for the temporal evolution of a droplet interface and succeeded in calculating the kinetics of shape evolution of a parallelepiped shape sample towards the equilibrium spherical shape. Basically, they used the following principles: If a perturbation was introduced at the interface with an initial amplitude *A*_0_, the amplitude of this perturbation decayed over time exponentially with a characteristic time *τ* as:

(4) $$A=A_{0}\;e^{-t/\tau }$$

If *C*_0_ is the equilibrium roundness of the sample (which will be defined later) at temperature *T*, in the presence of viscous flow roundness, *C* will increase and will evolve over time as follows:

(5) $$C=C_{0}-A_{0}\;e^{-t/\tau }$$

*τ* is a time constant for the decay that depends on the fluid properties of viscosity *η* and density ρ, the radius *R* of the initially unperturbed free droplet, and the oscillation mode *n*, with a constant value. Regardless of the value of *n*, *τ* is directly proportional to *η* [22]:

(6) $$\tau =\frac{\eta \left(n-1\right)\;\left(2n+1\right)}{\rho R^{2}}\;\propto \eta$$

Up unil now, we have not mentioned the influence of surface tension on the value of *τ*. In order to check the ratio between viscous effects and surface tension, a useful parameter is the Ohnesorge number, defined as in Equation (7) [22]:

(7) $$O_{h}=\frac{\eta }{\sqrt{2\rho \gamma R}}$$

where *η* is viscosity, *ρ* is density, *γ* is the surface tension coefficient, and *R* is a characteristic length, taken as the radius of the droplet to which the shape perturbation is introduced. When *O_h_* > 1, the viscous effect dominates over surface tension. In this experimental work, in all of the cases, *O_h_* has values over 100, thus clearly viscosity effects clearly dominate over surface tension, and this is the reason we do not consider *τ* as a function of surface tension. The surface tension values of the glasses used in this work range from 0.2 to 0.4 Jm^−2^ because of their different compositions and changes with temperature. If we analyze the experimental methods used up until now, basically they are based on Equation (1). An external shear stress is mechanically applied to the sample, through the interaction of a solid external surface with a glass surface, and the evolution of the sample deformation or a related quantity (penetration rate of a bar on the glass sample) is measured. This fact, requires that the external surface interacting with the glass surface be extremely well specified chemically and mechanically over all the measurement range and of course that no chemical reactions occur between them and even that no physical transformations be induced in the glass sample; for example, that the device surface could act as nucleation surface for crystallization. Of course, we cannot avoid glass thermodynamically favored transformations such as surface crystallization or glass–glass separation. In these cases, an effective viscosity value will be obtained. When non stoichiometric partial crystallization takes place, the viscosity value obtained corresponds to the mixture of the remaining glass with a chemical composition that corresponds to the remaining elements and crystals suspended in it. Sometimes, crystallization is so profuse that it inhibits the viscosity measurement. Glass–glass phase separation affects the overall viscosity and an experimental example will be given here.

Both *η* and *τ* are proportional to each other and depend on the glass chemical composition and temperature. *T* can be precisely measured in almost all laboratories, and analyzing sample pictures during their shape evolution, *τ* can also be determined easily. However, we need the viscosity value, because it is the quantity of scientific and technological interest. Thus, we can think of *η* as a function of *T* and τ.

(8) $$\eta =\eta \left(T,\tau \right)$$

In order to measure *τ*(*T*), a small sample with an irregular shape and about 0.003 m in characteristic length was heated up to temperature *T*, such that viscous flow occurred in a laboratory time scale and the particle evolved towards its minimum surface energy shape, namely towards a spherical shape. Temperature was maintained constantly during the spheroidization process. The shape evolution was recorded by taking pictures of the sample at regular time steps. At each time, the roundness of the sample image was calculated and, finally, from the evolution of roundness on *t*, a characteristic evolution time *τ* was obtained. We considered the initial particle shape mismatch with that of a sphere as a perturbation from the spherical minimum energy shape. In this work, no shear stress was applied externally, instead surface tension from the same sample external surface was used.

For a set of six different glasses, the *T* and *τ* values were measured during the isothermal shape evolution of irregular particles. For each measured *T*, *τ* pair data set, we added its corresponding assessed viscosity *η* value, obtaining three data sets *T*, *τ*, *η*. From these data sets, a mathematical master function *η* = *η*(*T*, *τ*) was built. *T*, *τ* values from two other glasses were measured (test glasses). Using the *η* = *η*(*T*, *τ*) function, we could assign viscosity values to the latter. The *η* values assigned to the test glasses using this master function were in good agreement with the available experimental data.

This method can be useful when, for example, no specific viscosity measurement devices are available, only very small sample quantities for example less than 1 cm^3^, or the sample is chemically corrosive.

Considering Equation (8), in this paper, we propose that two glasses that at a given *T* exhibit the same characteristic time *τ* during a given viscous flow process, have the same viscosity value. Or, in other words, if for any glass *T*, *τ* are known, we can assign a viscosity value through comparison with other glass of a known viscosity that exhibits the same *T*, *τ* values.

## 2. Materials and Methods

### 2.1. Materials

Eight glasses with the compositions detailed in Table 1 were used. All of the glasses were prepared with at least 99.9% purity chemicals from Sigma-Aldrich, St. Louis, MO, USA and platinum or porcelain crucibles.

*T*, *τ* measurements made on SiCaDF, WG, VG, SmAS, YAS, and SiTiFD glass samples were used to build the master function log(*η*) = function (*T*, log(*τ*)), which, from now on, for simplicity, we will refer to as *η* = *η*(*T*, *τ*). Measurements of *T*, *τ* on SG7 and VG98 samples were used to test the reliability of the previously built masterfunction *η* = *η*(*T*, *τ*).

### 2.2. Methods

#### 2.2.1. DTA Measurements

The glass transition temperature *T_g_* was measured for each glass using powder samples in a TAQ600 SDT Calorimeter. From each DTA measurement, the start temperatures of crystallization *T_s_* and the crystallization peak *T_x_* were also determined. The temperature range of applicability of the methodology proposed in this work is between *T_g_* and *T_s_*. Table 2 shows the obtained *T_g_*, *T_s_*, and *T_x_* values.

#### 2.2.2. VFT Parameters

The VFT parameters on glasses SiCaDF, WG, VG, SmAS, YAS, and SiTiFD were obtained for each glass by fitting Equation (2) to the *η* = *η*(*T*) data set calculated in Pa s with the software Sciglass 7.9, including *η* = 10^12.5^ Pa s at *T_g_*, which were measured in this work. For VG glass, the *T_g_* value was also taken from Sciglass software 7.9.

For SG7 and VG98 glasses, the experimental values were fitted with Equation (2), and *η* = 10^12.5^ Pa s at *T_g_* was also included. See Table 2.

#### 2.2.3. Hot Stage Microscopy Measurements: Determination of Roundness and *τ* Values

Above *T_g_*, and in the glass softening temperature range (when viscosity was in the range 10^9.9^–10^5.5^ Pa s), we were able to record the deformation kinetics at fixed temperatures and to follow the roundness [24] of the sample, using the software included in the MISURA device. Each picture was analyzed by MISURA software Version 3.32, calculating the sample perimeter P and area A as a percentage of their initial values. Accordingly, the roundness C was calculated as C = 4π A/(P^2^).

At each *T*, the shape of an irregular particle sample of a typical size of about 3 × 3 × 3 mm^3^ was followed using a hot stage MISURA HSM microscope. We used a 25 K/min heating ramp up to 40 K below the plateau temperature and 10 K/min approaching the plateau temperature. This procedure was used in order to minimize deformation before reaching the plateau temperature and also to minimize overheating.

**Figure 2:**
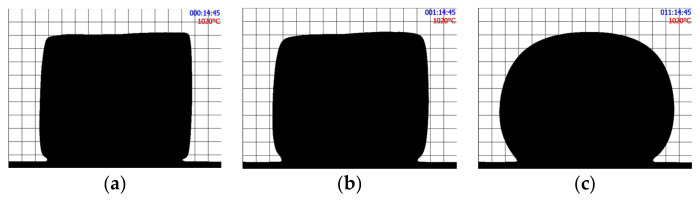
SmAS cubic glass sample heated at 1293 K. (**a**) At the beginning of the thermal plateau. (**b**) After 1 h at 1293 K. (**c**) After 11 h. The change in shape over 11 h is recorded and roundness on time is determined.

**Figure 3:**
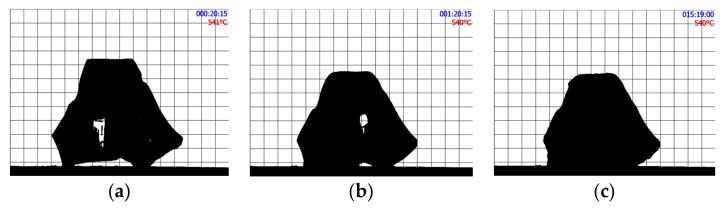
SiTiFD irregular glass sample heated at 813 K. (**a**) Just at the beginning of the thermal plateau. (**b**) After 1 h at 813 K. (**c**) After 15 h. The slight change in shape over 15 h is recorded as roundness on time, as shown in Figure 5. During heat treatment pictures, were taken every 15 s.

**Figure 4:**
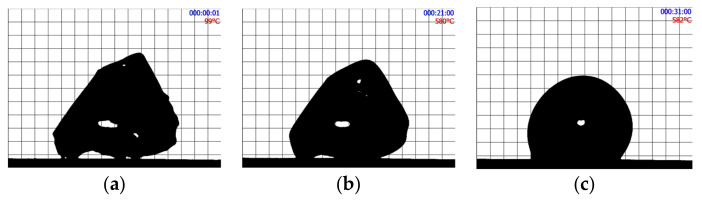
SiTiFD irregular glass sample heated at 853 K. (**a**) Before reaching the thermal plateau. (**b**) At the beginning of the thermal plateau at 853 K. (**c**) After 10 min. The fast change in shape is recorded as roundness on time, as shown in Figure 5. During heat treatment, pictures were taken every 15 s.

**Figure 5:**
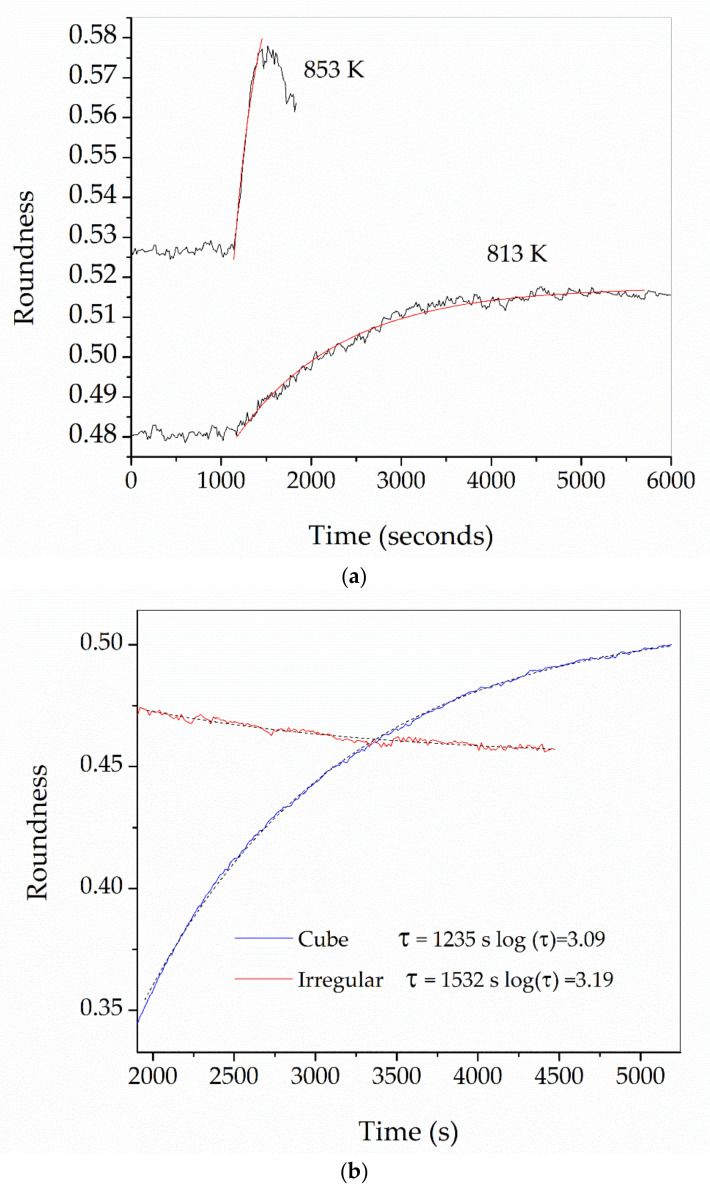
(**a**) Roundness vs. time, measured on two irregular SiTiFD glass samples, as shown in Figure 3 and Figure 4. The sample heated at 813 K exhibits a slight viscous flow, increasing its roundness. The sample heated at 853 K has faster kinetics and after a few minutes reaches its almost spherical shape. In red solid lines: fittings to both measured curves. Characteristic times *τ* obtained from both curves by fitting Equation (5) are: Log(τ) at 813 K = 3.08 (*τ* in s), log(τ) at 853 K = 2.43 (*τ* in s). (**b**) Comparison of relaxation times measured on WG samples at 1023 K using cube geometry (blue line) and irregular geometry (red line). The dashed black lines are Equation (5) fittings.

Figure 2 shows the shape evolution of an initially cubic SmAS glass sample at 1293 K: just at the beginning of the plateau, after 1 h, and after 15 h. Figure 3 and Figure 4 show a set of three pictures each, obtained from initially irregular shape samples of SiTiFD glass at 813 K and 853 K, respectively. Actually, sample pictures were taken at regular time steps, accumulating a total of about 2000 pictures for each experiment. For each picture, a roundness value was available from the software, and these values are shown in Figure 5; consequently, *τ* values were obtained. The results are shown in Table 3. As shown in Figure 5a, Equation (5) was fitted to the roundness evolution of each glass sample and each temperature, obtaining characteristic times *τ*.

We compared the values of the characteristic times *τ* obtained using initial cubic samples or initial irregular samples. For the same glass and identical thermal processes, we obtained similar *τ* values (See Figure 5b), thus we used any indistinct initial sample shape.

Experimental errors in T(K): For Log(τ) ~2 (*τ* in s), the error is ~ ± 10 K, and for Log(τ) >2 (*τ* in s), the error is ~ ± 1 K. Experimental errors in Log(τ) ~ 0.1 (*τ* in s). Errors in Log(*η*) are not reported by the software used; different models typically differ by ±0.75 (*η* in Pa s).

## 3. Results and Discussion

### 3.1. DTA Measurements and VFT Parameters

**Figure 6:**
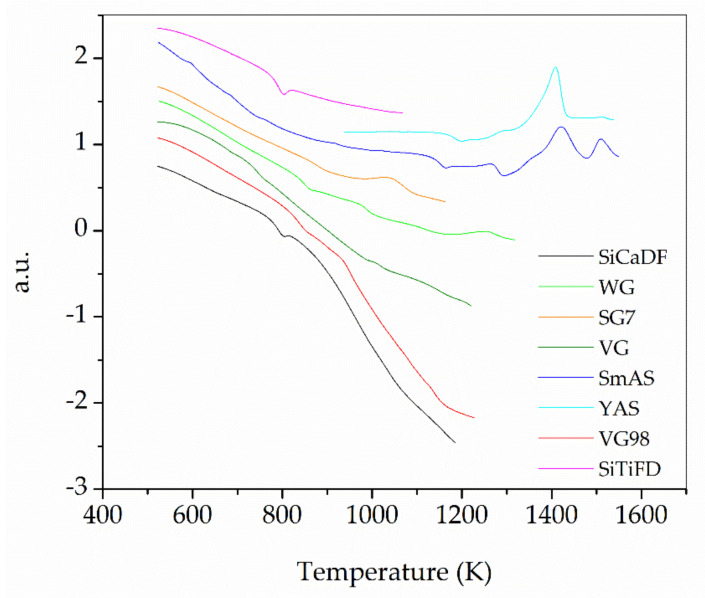
DTA traces measured for all of the glasses in this work. Lines were translated vertically to avoid crossings over each other.

As indicated in Section 2.2.1, the glass transition temperature values *T_g_*, start temperature of crystallization *T_s_*, and crystallization peak temperatures *T_x_* for each glass were determined from the DTA measurements. DTA traces are presented in Figure 6. *T_g_*, *T_s_*, and *T_x_* values can be found in Table 2.

**Figure 7:**
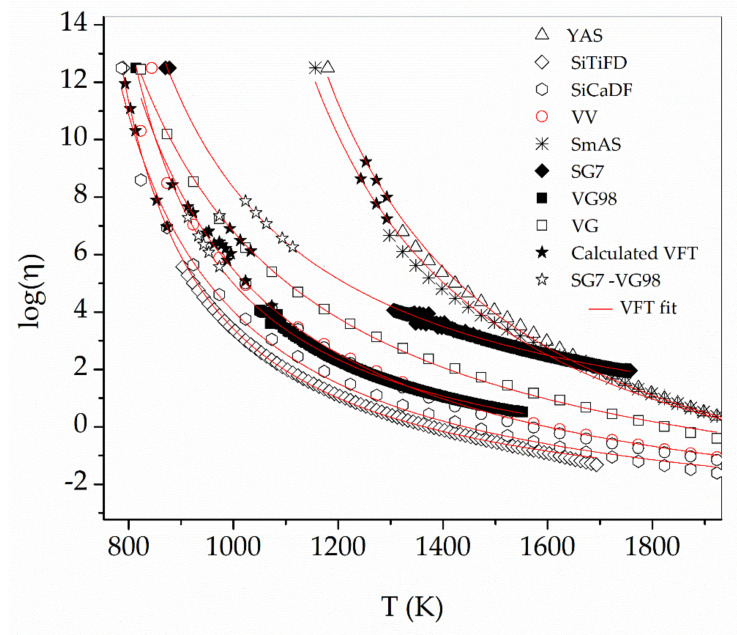
*T*, *τ*, *η* values presented in Table 2 and used to build the master function (solid stars). Expected viscosity values for test glasses SG7 and VG98 (hollow stars). Region of validity of this method (region where stars are located).

Figure 7 shows the viscosity data obtained from SciGlass 7.9, except for SG7 and VG98, which are from the experimental data. From the VFT fittings to these data, parameters log(*η*_0_), B, and T_0_ have been determined and are shown in Table 2.

### 3.2. T,τ,η Data Sets

In Table 3, we present *T*, *τ*, *η* sets for six glasses used to build the master function *η* = *η*(*T*, *τ*).

### 3.3. Master Function η = η (T,τ)

**Figure 8:**
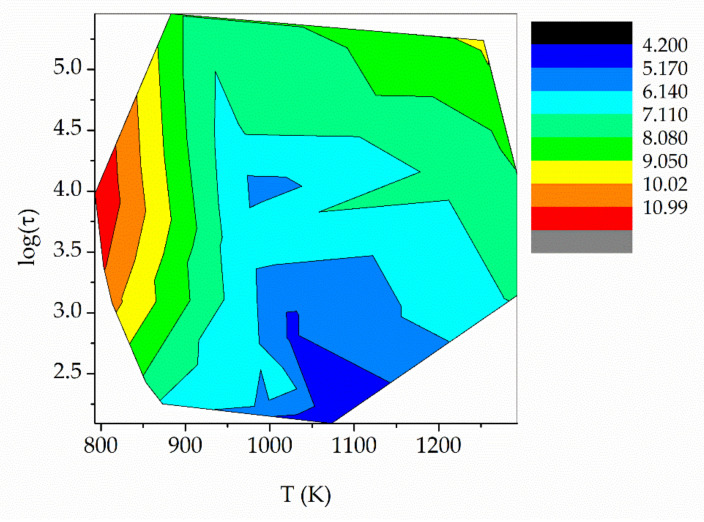
Iso-viscosity lines as a function of *T* and log(*τ*). *τ* in s, viscosity in Pa s.

When we plot log(*η*) (*η* in Pa s) contour lines as a function of independent variables *T* (K) and log(*τ*) (*τ* in s), we find a pattern, which is shown in Figure 8. Such a pattern suggests that it is possible to fit a mathematical function for *η*(*T*, *τ*). After many trials, we fitted the function shown in Equation (9).

(9) $$log\left(\eta \left(T,\tau \right)\right)=\frac{A}{T}+B+C*T+D*log\left(\tau \right)+E\;*T*log\left(\tau \right)+F*T^{2}+G*{\left(log\left(\tau \right)\right)}^{2}$$

Actually, this is an estimated equation where the first terms account for an Arrhenius type behavior and the following terms account for deviations from this Arrhenius type behavior. After disregarding the terms with less impact on the *η*(*T*, *τ*) result, we obtained Equation (10):

(10) $$log\;\left(\eta \left(T,\tau \right)\right)=\frac{184069}{T}-474.7+0.41377*T-1.01323*log\left(\tau \right)+0.0016*T*log\left(\tau \right)-0.00011896*T^{2}$$

with a regression coefficient of R = 0.924. The error associated at each constant is 40%, except for D which is 80%. The average disagreement in log(*η*) is 0.4 (*η* in Pa s) and σ = 0.3.

**Figure 9:**
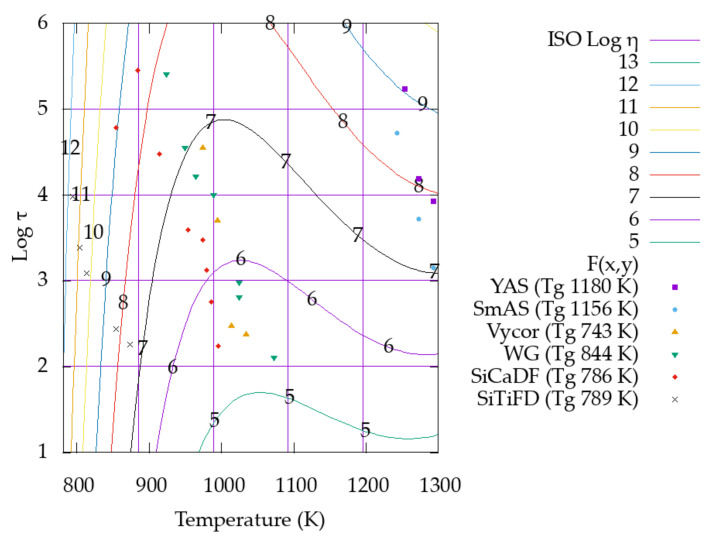
*T*, log(*τ*) experimental data points and iso-viscosity lines from the master function *η* = *η*(*T*, *τ*) built in this section. It is possible to observe that *T*, *τ* data for the same glass is along a line and *T_g_* increases from left to right. *τ* in s, viscosity in Pa s.

The data presented in Table 3 are plotted in Figure 9. In Figure 9, it can be seen that the set of *T*, *τ* points corresponding to each glass are ordered approximately along a straight line, with a similar slope for the different glasses.

In addition, it can be seen that these lines of points are ordered according increasing T_g_ values from left to right within the error of measurement of the *T_g_* values. The slope ∂log(*τ*)/∂*T* corresponding to a set of measurements for one glass cannot be lower than the slope of the iso-viscosity lines in Figure 9 (see Section 3.3). A lower slope value would indicate an increase in viscosity with the increase in temperature, which is not physically expected.

**Figure 10:**
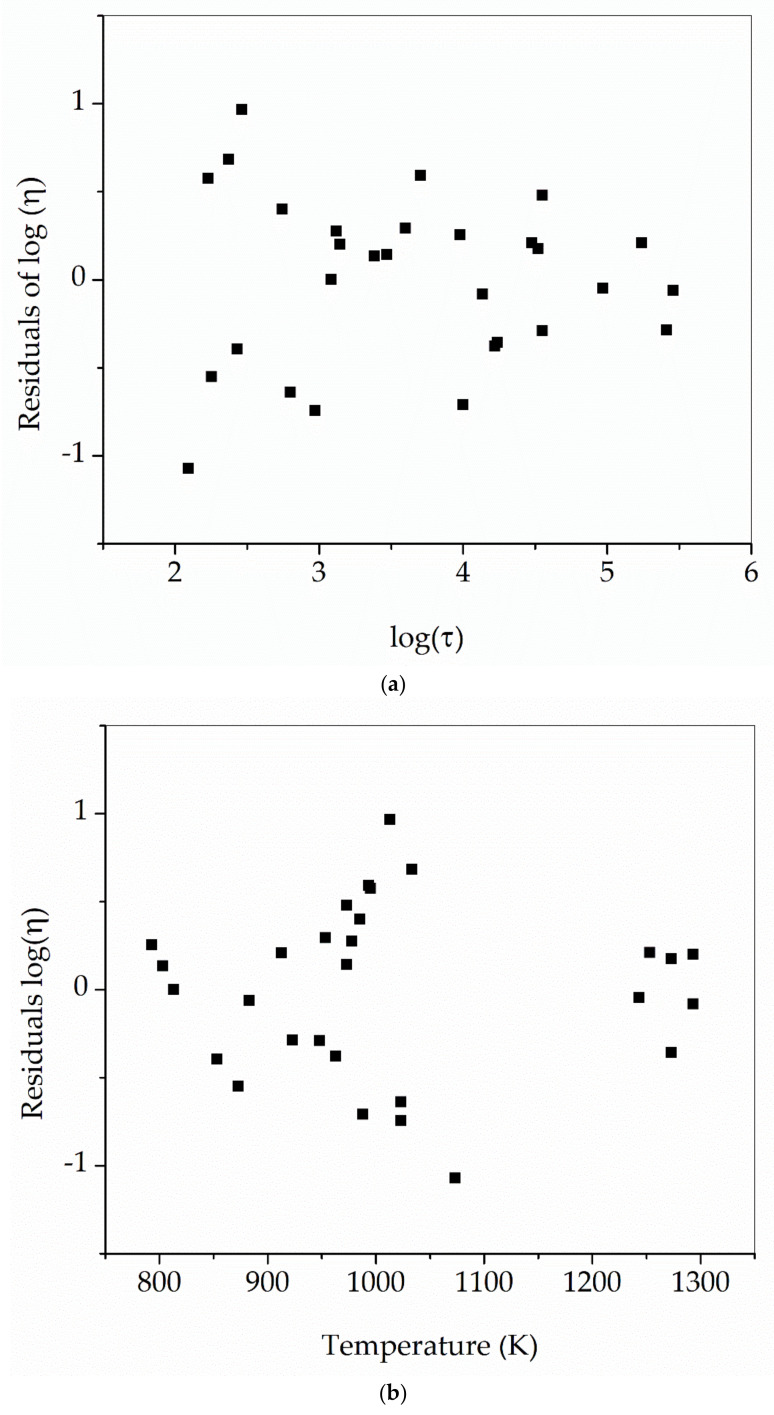
Residuals of the fitted function *η* = *η*(*T*, *τ*). In (**a**), it is found that residuals increase with the decreasing value of τ. In (**b**), no correlation is seen between residuals and temperature. *η* in Pa s and *τ* in s.

Figure 10 shows the residuals of the master function with the fitted data. Although there is not a direct correlation with temperature, larger errors are found for shorter *τ* values.

### 3.4. Reliability Test of the Master Function

In order to test the reliability of *η* = *η* (*T*, *τ*), we measured *T*, *τ* for two different silicate glasses: VG98 and SG7. The obtained *T*, *τ* data are presented in Table 4. Using Equation (10), we assigned *η* values to each *T*, *τ* pair. These assigned values are listed in Table 4 as (#).

**Figure 11:**
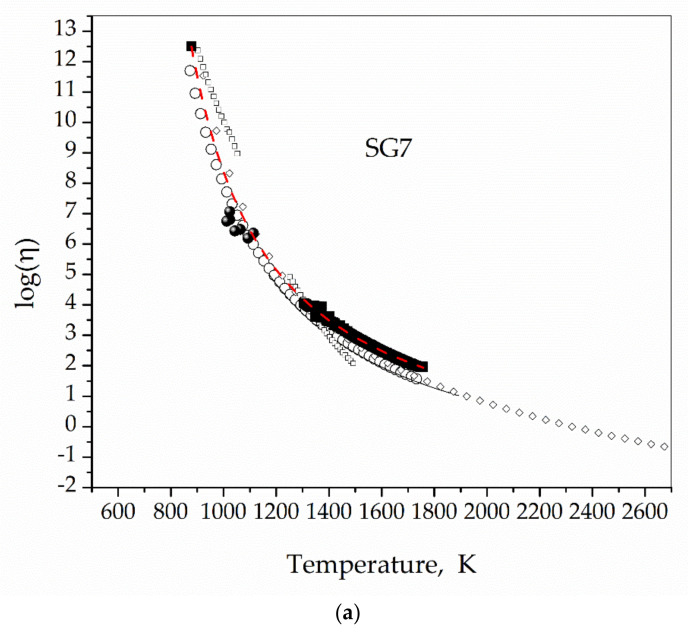

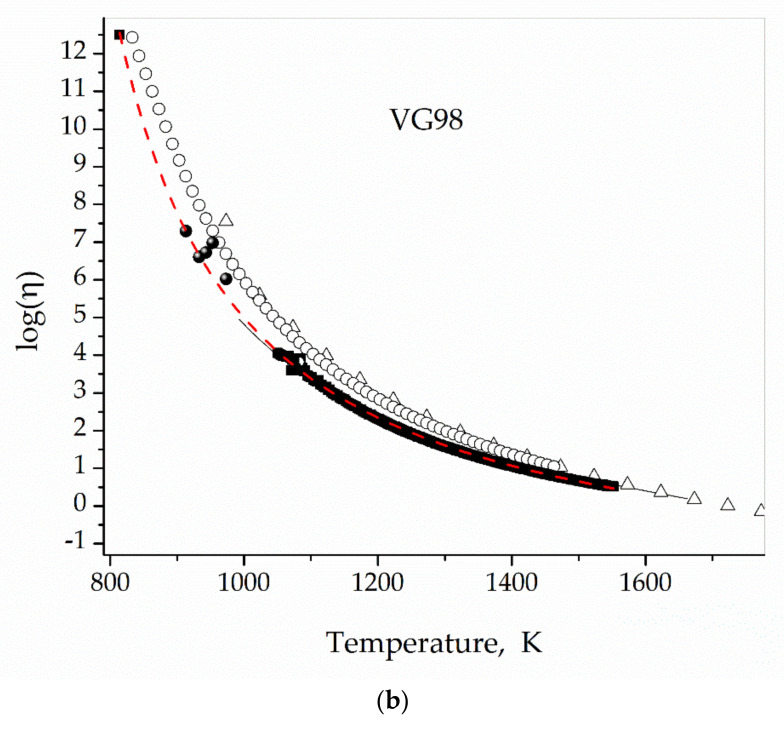
Assigned viscosity values for SG7 (**a**) and VG98 (**b**) glasses (solid spheres), measured with the rotating concentric cylinders technique (solid black squares), VFT fit to measured values and *η*(*T_g_*) = 10^12.5^ Pa s (Red dashed lines), theoretical values calculated using SciGlass 7.9 (hollow circles, squares, triangles, and diamonds). Viscosity in Pa s.

In column (^), the expected viscosity values according the respective VFT functions are presented. We found an average disagreement percentage of 8% and errors lower than 14%. In Figure 11a,b we show the agreement of the assigned viscosity values with viscosity values calculated using SciGlass 7.9 and our previous measurements presented in Table 4.

In short, we built a function *η* = *η*(*T*, *τ*) being *T* the temperature and *τ* an experimental characteristic time of our experiment: the spheroidization of an irregular particle evolving by viscous flow towards a new shape. *T*, *τ* are measurable quantities. *T* has a value chosen between *T_g_* and the temperature where crystallization starts *T_s_*. *τ* depends on the glass viscosity and surface tension through the glass chemical composition, as well as on temperature. Then, Equation (8) is a transcendental equation in *η*, which we is not solved analytically. However, using calculated values for *η* (we do not have available measured values, but they could be found in the future), we found that *η* = *η*(*T*, *τ)* was a single-valued function of *T*, *τ*. In other words, two glasses with the same value of *T*, *τ* had the same viscosity at temperature *T*.

Another remarkable finding is that, contrary to what occurs with structural relaxation where an average relaxation time is used, in our case, an exponential behavior with only one characteristic time applied.

To the best of our knowledge, no method that allowed for reasonably assigning viscosity values measuring the rounding kinetics at a fixed temperature of an irregular sample have been presented up until now. A small volume of samples of about 0.05 cm^3^ were used in each measurement. Glass does not need to be translucid. No special sample geometry is needed, the only condition is that the sample geometry must not be spherical and should preferably be irregular. Small pieces obtained by cracking a larger piece were used in this work.

For six silicate glasses we measured the *T*, *τ* values. For high temperatures, overheating needed to be minimized. Errors in *τ* increased with a decrease in *τ*. With these *T*, *τ* values, we built *T*, *τ*, *η* sets. *η* values were calculated with the Priven 2000 theoretical model available in the software SciGlass 7.9. After that, we fitted a master function *η* = *η*(*T*, *τ).*

For the remaining two silicate glasses, we measured *T*, *τ* values, and the viscosity value *η* was assigned to each *T*, *τ* using the previously built master function. When we plotted these *η* values assigned for both test glasses together with their available experimental and theoretical values, we found that they agreed well. Thus, within the *T*, *τ* domain determined by our measurements on six glasses, we conclude that for other glasses of an unknown viscosity, we could assign a viscosity value at temperature *T*, using the master function presented here, if we measured *T*, *τ*.

The data set used here for building *η* = *η*(*T*, *τ*), may be enriched with new experimental data and a better master function could be obtained; moreover, the application limits should be expanded. The actual validity region for this method is that delimited by the experimental points (solid stars) in Figure 7. We also tested its reliability with two other borosilicate glasses and found that the viscosity values assigned were in agreement with the measured and calculated values. Although not presented in this work, attempts to apply this method to 45S5 glass were not successful because crystallization inhibited the viscous flow [25]. All of the measurements in this work were done under air, it could be of interest to check the effect of different gaseous atmospheres on these results.

## 4. Conclusions

We can assign a viscosity value to glass at temperature *T*, in the softening temperature range, if we measure two quantities: the characteristic deformation time *τ* and the temperature *T* at which the deformation takes place. This is achieved using the master function *η* = *η*(*T*, *τ*) proposed and built in this work (Equation (10)) with *T*, *τ*, *η* values measured (*T* and *τ*) and calculated (*η*) for six different glasses. This master function can be improved by users by adding new *T*, *τ*, *η* measured data.

## Figures and Tables

**Table 1:** Glass compositions mol%. (*) nominal, (#) as determined in this work using EDS. Glasses are ordered according to decreasing silica content.

	SiCaDF (*)	WG (#)	SG7 (#)	VG (#)	SmAS (#)	YAS (#)	VG98 (#)	SiTiFD (*)
SiO_2_	73.3	72.6	71.7	68.8	59.8	58.1	56.7	32.3
B_2_O_3_			8.3	24.8			12.4	4.9
Al_2_O_3_		2.1	8.6	0.4	24.7	20.1	2.6	2.4
TiO_2_								25.0
Li_2_O	2.6							
Na_2_O	10.8	13.1	7.4	6.0			17.5	32.3
CaO	9.8	5.9	2.7				4.1	
BaO								1.0
MgO	3.5	5.6	1				2.1	
Fe_2_O_3_		0.2						
Y_2_O_3_						21.7		
Sm_2_O_3_					15.5			
K_2_O		0.5						
ZrO								1.0
P_2_O_5_								0.1
MoO_3_								1.2

**Table 2:** From the DTA measurements (this work): *T_g_* values, temperature of crystallization start *T_s_*, and crystallization peak *T_x_* for each glass. (*): slight crystallization is observed above *T_g_*. From the VFT fittings (this work): parameters log(*η*_0_), B, and T_0_. In brackets: measurement or fitting errors in last digits.

Glass	*T_g_* (K)	*T_s_* (K)	*T_x_* (K)	log(*η*_0_)	B	*T*_0_ (K)
SiCaDF	786 (10)	1266	1300	−3.40 (2)	5229 (52)	441 (4)
WG (*)	844 (8)	1143	1257	−4.6 (2)	4908 (384)	516 (23)
SG7 (*)	878 (7)	953	1037	0.36 (4)	4212 (50)	580 (3)
VG	742 (10)	986	1009	−7.3 (3)	9112 (572)	321 (28)
SmAS	1156 (10)	1340	1419	−5.1 (1)	6054 (176)	802 (10)
YAS (*)	1180 (7)	1309	1409	−5.3 (3)	6342 (141)	818 (8)
VG98 (*)	814 (8)	899	933 (*)	−0.58 (2)	2817 (19)	628 (2)
SiTiFD	789 (5)	1209	1301	−3.51 (5)	2600 (41)	625 (3)

**Table 3:** T, log(*τ*) measured using high temperature microscopy, and log(*η*) calculated using VFT functions shown in Table 2. *T*, *τ*, *η* values to build the masterfunction *η* = *η*(*T*, *τ*).

Glass	*T* (K)	Log(*τ*) (*𝛕* in s)	Log(*η*) (*η* in Pa s)
SiCaDF	883	5.457	8.430
SiCaDF	913	4.475	7.678
SiCaDF	985	2.745	6.212
SiCaDF	978	3.119	6.337
SiCaDF	953	3.599	6.812
SiCaDF	973	3.469	6.428
SiCaDF	995	2.230	6.038
VV	923	5.409	7.455
VV	948	4.550	6.757
VV	963	4.220	6.376
VV	988	4.000	5.795
VV	1023	2.970	5.078
VV	1023	2.800	5.078
VV	1073	2.093	4.209
VG	1033	2.370	6.120
VG	1013	2.465	6.496
VG	993	3.705	6.910
VG	973	4.549	7.348
SmAS	1273	4.237	7.767
SmAS	1243	4.970	8.643
SmAS	1293	3.143	7.243
YAS	1293	4.133	8.002
YAS	1273	4.520	8.59
YAS	1253	5.238	9.232
SiTiFD	873	2.253	6.970
SiTiFD	853	2.430	7.890
SiTiFD	813	3.083	10.310
SiTiFD	793	3.980	11.950
SiTiFD	803	3.382	11.080

**Table 4:** *T* (K), log(*τ*(*T*)) (s), log(*η*) (Pa s) data. (*) Measured in this work; (^) Calculated using VFT in Table 2. (#) Assigned using Equation (10). (%) Percentage difference between # and ^.

Glass	*T* (K) (*)	Log(*τ*) (*) (*τ* in s)	(^) Log(*η*) (*η* in Pa s)	(#) Log(*η*) (*η* in Pa s)	%
SG7	1023	4.458	7.862	6.802	−14
SG7	1043	3.807	7.452	6.428	14
SG7	1063	3.790	7.076	6.482	−9
SG7	1093	3.190	6.567	6.189	−6
SG7	1113	3.266	6.259	6.350	−1
VG98	913	3.960	7.294	7.292	0
VG98	933	3.960	6.647	6.980	−5
VG98	943	3.690	6.354	6.724	−6
VG98	953	3.660	6.08	6.602	−9
VG98	973	2.880	5.578	6.018	−8

## Data Availability

All experimental data published in this work can be requested to their authors by email.
